# The circadian clock protein Rev-erbα provides neuroprotection and attenuates neuroinflammation against Parkinson’s disease via the microglial NLRP3 inflammasome

**DOI:** 10.1186/s12974-022-02494-y

**Published:** 2022-06-06

**Authors:** Liang Kou, Xiaosa Chi, Yadi Sun, Chao Han, Fang Wan, Junjie Hu, Sijia Yin, Jiawei Wu, Yunna Li, Qiulu Zhou, Wenkai Zou, Nian Xiong, Jinsha Huang, Yun Xia, Tao Wang

**Affiliations:** 1 Department of Neurology, Union Hospital, Tongji Medical College, Huazhong University of Science and Technology, Wuhan, 430022 China; 2 Department of Neurology, The First Affiliated Hospital of USTC, Division of Life Sciences and Medicine, University of Science and Technology of China, Hefei, Anhui China

**Keywords:** Parkinson’s disease, Circadian rhythm, Rev-erbα, NLRP3, Neuroinflammation

## Abstract

**Background:**

Circadian disturbance is a common nonmotor complaint in Parkinson’s disease (PD). The molecular basis underlying circadian rhythm in PD is poorly understood. Neuroinflammation has been identified as a key contributor to PD pathology. In this study, we explored the potential link between the core clock molecule Rev-erbα and the microglia-mediated NLR family pyrin domain-containing 3 (NLRP3) inflammasome in PD pathogenesis.

**Methods:**

We first examined the diurnal Rev-erbα rhythms and diurnal changes in microglia-mediated inflammatory cytokines expression in the SN of MPTP-induced PD mice. Further, we used BV2 cell to investigate the impacts of Rev-erbα on NLRP3 inflammasome and microglial polarization induced by 1-methyl-4-phenylpyridinium (MPP^+^) and αsyn pre-formed fibril. The role of Rev-erbα in regulating microglial activation via NF-κB and NLRP3 inflammasome pathway was then explored. Effects of SR9009 against NLRP3 inflammasome activation, microgliosis and nigrostriatal dopaminergic degeneration in the SN and striatum of MPTP-induced PD mice were studied in detail.

**Results:**

BV2 cell-based experiments revealed the role of Rev-erbα in regulating microglial activation and polarization through the NF-κB and NLRP3 inflammasome pathways. Circadian oscillation of the core clock gene Rev-erbα in the substantia nigra (SN) disappeared in MPTP-induced PD mice, as well as diurnal changes in microglial morphology. The expression of inflammatory cytokines in SN of the MPTP-induced mice were significantly elevated. Furthermore, dopaminergic neurons loss in the nigrostriatal system were partially reversed by SR9009, a selective Rev-erbα agonist. In addition, SR9009 effectively reduced the MPTP-induced glial activation, microglial polarization and NLRP3 inflammasome activation in the nigrostriatal system.

**Conclusions:**

These observations suggest that the circadian clock protein Rev-erbα plays an essential role in attenuating neuroinflammation in PD pathology, and provides a potential therapeutic target for PD treatment.

**Supplementary Information:**

The online version contains supplementary material available at 10.1186/s12974-022-02494-y.

## Introduction

Circadian dysfunction is one of the most common nonmotor symptoms of Parkinson’s disease (PD) and affects about 64% of PD patients [1]. It is typically characterized by sleep disturbances and by disruptions in motor activity, autonomic function, and the responsiveness to dopaminergic treatments [2, 3]. Circadian abnormalities in elderly adults are associated with an increased PD risk [4]. In generally, circadian disturbance often occurs decades before the onset of motor symptoms, making it a key factor in poor life quality [4, 5]. Studies have shown that the core clock genes of PD patients are disturbed during the early stage of the disease [6], and interference with the circadian rhythm or core clock genes in animal models of PD leads to exacerbated motor symptoms and increased loss of dopaminergic neurons [7, 8]. However, the causal mechanism underlying circadian disturbance in PD are still poorly understood.

Microglia-mediated neuroinflammation is considered as an important pathological feature of PD [9]. Microglial overactivation by harmful stimuli such as lipopolysaccharide (LPS), toxic misfolded proteins may shift these cells from anti-inflammatory state to a proinflammatory state, with release of proinflammatory cytokines such as Interleukin-1β (IL-1β) and tumor necrosis factor-α (TNF-α), leading to further neuronal injury [10]. The NLR family pyrin domain-containing 3 (NLRP3) inflammasome, an important component of the innate immune system, has been increasingly associated with occurrence and progression of PD pathogenesis in PD models and PD patients [11–14]. Exome sequencing analysis of human NLRP3 gene variants revealed that multiple single-nucleotide polymorphisms were associated with a significantly reduced risk of PD development [15]. Moreover, inhibiting the NLRP3 inflammasome can reduce α-synuclein deposition, alleviate dopaminergic neuron damage and improve motor function [11, 16, 17].

There is growing evidence that circadian rhythm and neuroinflammation are closely linked. Studies have reported that astrocyte activation is autonomously regulated by the clock gene Bmal1, and behavioral circadian interference or manipulation of Bmal1 can induce astrocyte hyperplasia and dysfunction, along with oxidative stress, synaptic damage and increased inflammation in the CNS [18, 19]. Another study showed that the loss of Bmal1 or Rev-erbα leads to the upregulation of the complement genes C4b and C3 in astrocytes, microglia activation, and increased synaptic phagocytosis [20]. In 2-month-old amyloid precursor protein knock-in (APP-KI) mice, the Clock/Bmal1-driven negative feedback loop of transcription in microglia was impaired, and activation of Rev-erbα promoted the expression of inflammatory cytokines and cognitive impairment [21]. In contrast, some researches have shown that Rev-erbα activation has an inhibitory effect on neuroinflammation [22, 23]. More interestingly, the link between the circadian clock Rev-erbα and neuroinflammation in PD has been reported, as Rev-erbα deficiency exacerbates 6-OHDA-induced dopaminergic neurodegeneration, possibly related to microglial proliferation in substantia nigra (SN) [24].

Since Rev-erbα is closely associated with neuroinflammation in the CNS, and Rev-erbα ablation exacerbates pathological changes in PD, we hypothesized that Rev-erbα was an important factor that mediated the pathological progression of PD. To test this hypothesis, we first constructed the MPTP-induced PD model, and evaluated the expression patterns of Rev-erbα, microglial activation and the expression of inflammatory cytokines. The effects of the Rev-erbα-specific small molecule agonist SR9009 on dopaminergic neurons in MPTP mouse were also examined. Furthermore, we examined the effect of SR9009 on glial hyperplasia, microglial polarization and NLRP3 inflammasome activation. Lastly, the potential mechanism by which Rev-erbα regulates microglia-mediated neuroinflammation induced by 1-methyl-4-phenylpyridinium (MPP^+^) or αsyn pre-formed fibril (αsyn PFF) was further investigated in vitro.

## Materials and methods

### Animals

Two-month-old C57BL/6J male mice were purchased from Beijing Vital River Laboratory Animal Technology Co. Ltd., and housed on a 12/12 h light/dark cycle (light–dark, 07:00 light on, 19:00 light off) with food and water ad libitum. All animal experiments were carried out in accordance with the guidelines of the Animal Care and Use Committee of Maximum Efforts of Huazhong University of Science and Technology (HUST).

### Experimental design

**Fig. 1:**
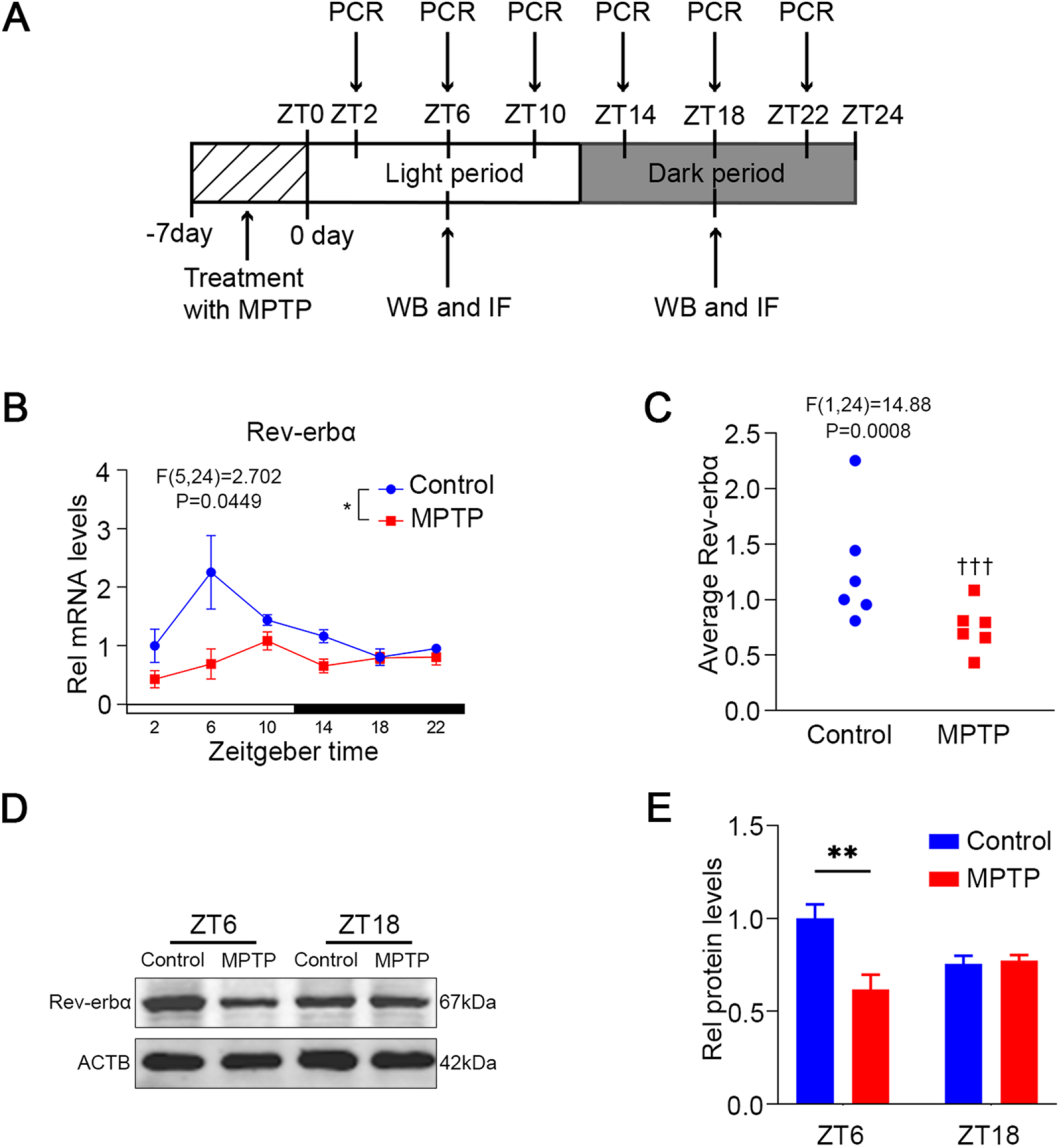
Aberrant diurnal Rev-erbα rhythm in the SN of MPTP-induced Mice. **A** Schematic diagram of expression profiles of Rev-erbα. After 7-day continuous injection of MPTP (25 mg/kg) or 0.9% saline, mice were euthanized at 4-h intervals throughout the day at ZT2, 6, 10, 14, 18, 22. **B** The mRNA level of Rev-erbα in the SN was quantified using real-time PCR. (*p < 0.05, two-way ANOVA test, interaction between time and genotypes). **C** The average values of Rev-erbα over the course of the day were calculated. (^†††^*p* < 0.001, two-way ANOVA test, interaction between genotypes). A representative western blot image (**D**) and the statistical graph (**E**) of Rev-erbα expression at ZT6 and ZT18 in the SN. The protein level of Rev-erbα was normalized to β-actin. (^##^*p* < 0.01, two-way ANOVA test). *n* = 3–5 for each time point. Data were presented as mean ± SEM

First, expression patterns of Rev-erbα and morphological changes in microglia in the SN in control (*n* = 36) and MPTP mice (*n* = 36) were investigated using immunoblotting, immunofluorescence and RT-PCR. Mice in the experimental group received a single intraperitoneal injection of MPTP (25 mg/kg; Sigma) every day for 7 consecutive days. Control mice were given an equal amount of saline at the same time. Mice were harvested the day after the last MPTP injection, and euthanized at 4-h intervals throughout the day (Fig. 1A).

Then, the effects of SR9009 on motor function, dopaminergic neuron loss, glial hyperplasia, microglial polarization, the expression of inflammatory cytokines and NLRP3 inflammasome activation in the MPTP mouse model were examined. Forty mice were randomly assigned to four groups: saline group, SR9009 group, MPTP group, and MPTP + SR9009 group. SR9009 was injected intraperitoneally (100 mg/kg/day) for 7 consecutive days prior to MPTP administration and then continued along with MPTP treatment (Fig. 6A). SR9009 was administered daily at ZT8, followed by MPTP 1 h later. Behavioral tests began the day after completing 14 days of SR9009 treatment, and mice were killed after behavioral test (day 18).

### Behavioral assays

#### Balance beam test

The day after the completion of the 14-day SR9009 treatment, balance beam tests were examined. The balance beam was 0.5 cm wide, 1 m long and 40 cm high. A dark box was placed at the end of the balance beam for the mice to rest. The mice were retested three times in the afternoon with an interval of at least 15 min between tests. During the test, the time each mouse took to cross the balance beam was recorded.

#### Pole test

The day after the completion of the 14-day SR9009 treatment, pole test was examined. The animal was placed face-up near the top of a rough wooden pole (15 mm in diameter and 40 cm in height), and the time it took to reach the floor was recorded. After 3 days of learning adaptation (3 training sessions per day), each mouse was finally administered 3 formal tests.

#### Rotarod test

The day after the completion of the 14-day SR9009 treatment, rotarod tests were examined. The accelerated rotarod test was performed over 4 consecutive days with 3 days of training and acclimatization. The rotarod device was used for 3 tests per day at 5–40 rpm for 5 min. There was a break of more than 30 min between each test. The time for each mouse to fall was recorded, and 300 s were recorded for those who did not fall after more than 5 min. Each mouse was tested three times, and the results were then averaged.

### BV2 microglial cell culture and treatment

BV2 microglia were cultured in DMEM/high glucose medium containing 10% fetal bovine serum. The cells were kept at 37 °C in a humidified incubator with 5% CO_2_.

To induce inflammasome activation, BV2 cells were primed with MPP^+^ (200 uM, Sigma, USA) for 24 h. Alternatively, inflammasome activation was also induced by treatment with sonicated αsyn PFF (5 ug/ml, donated by Zhang’s Laboratory [25]) for 6 h. To examine the effects of Rev-erbα, NF-κB and NLRP3 inflammasomes on microglia, cells were pretreated with SR9009 (MedChem Express, USA), SR8278 (MedChem Express, USA), JSH-23 (MedChem Express, USA), or MCC950 (MedChem Express, USA) for 1 h, and then treated with MPP^+^ or aggregated αyn PFF for the indicated dose and time.

### Quantitative real-time PCR (qRT-PCR)

Total RNA was extracted by using TRIzol reagent (Takara, Japan) according to the manufacturer’s instructions. After the RNA was reversely transcribed into cDNA, qRT-PCR was carried out. qRT-PCR was performed on a StepOne Plus system (Applied Biosystems) and analyzed using StepOne 2.3 software. The experimental operating conditions were 95 °C for 5 min, 95 °C for 5 s and 60 °C for 30 s for 40 cycles. ACTB served as an internal standard. The primers used for PCR are shown in Table 1.

**Table 1:** Mouse primer sequences for quantitative real-time PCR (qPCR)

Gene	Forward (5ʹ-3ʹ sequence)	Reverse (3ʹ-5ʹ sequence)
*Rev-erbα*	TTTTTCGCCGGAGCATCCAA	ATCTCGGCAAGCATCCGTTG
*IL-1β*	AATGCCACCTTTTGACAGTGATG	AGCTTCTCCACAGCCACAAT
*IL-18*	TCAAAGTGCCAGTGAACCCC	GGTCACAGCCAGTCCTCTTAC
*IL-6*	ATCCAGTTGCCTTCTTGGGACTGA	TAAGCCTCCGACTTGTGAAGTGGT
*TNF-α*	AGC AAA CCA CCA AGT GGA GGA	GCT GGC ACC ACT AGT TGG TTG T
*Nlrp3*	ATTACCCGCCCGAGAAAGG	TCGCAGCAAAGATCCACACAG
*Arg-1*	TGCTCACACTGACATCAACACTCC	TCTACGTCTCGCAAGCCAATGTAC
*iNOS*	GGC AAA CCC AAG GTC TAG GTT	TCG CTC AAG TTC AGC TTG GT
*Actin*	GCCTCACTGTCCACCTTCCA	AGCCATGCCAATGTTGTCTCTT

### Protein isolation and western blot

RIPA lysis buffer containing phosphorylase inhibitors, cocktails, and PMSF was used to digest tissue for protein extraction. Western blotting was performed as described previously [26]. The following primary antibodies were used: rabbit anti-NR1D1 (ab174309, Abcam), rabbit anti-NR1D1 (14506-1-AP, Proteintech), rabbit anti-tyrosine hydroxylase (TH) (25859-1-AP, Proteintech), rabbit anti-IBA1 (10904-1-AP, Proteintech), mouse anti-GFAP (60190-1-Ig, Proteintech), rabbit anti-NF-κB p65 (#8242, Cell Signaling Technology), rabbit anti-phospho-NF-κB p65 (#3033, Cell Signaling Technology), rabbit anti-NLRP3 (BA3677, BOSTER), mouse anti-ASC (sc-514414, Santa Cruz Biotechnology), rabbit anti-caspase-1 (22915-1-AP, Proteintech), rabbit anti-IL-1β (A16288, ABclone), rabbit anti-IL6 (A0286, ABclone), mouse anti-actin (66009-1-Ig, Proteintech), mouse anti-TNF Alpha (60291-1-Ig, Proteintech), rabbit anti-iNOS (22226-1-AP, Proteintech), rabbit anti-IL-18 (10663-1-AP, Proteintech), mouse anti-Arginase-1 (66129-1-Ig, Proteintech), rabbit anti-CD163 (16646-1-AP, Proteintech), rat anti-CD68 (MCA1957, BIO-RAD), Anti-Alpha-synuclein (ab138501, Abcam).

### Immunofluorescence

Immunofluorescent stain was performed on paraffin-embedded sections or frozen sections of brain tissue. Paraffin sections were dewaxed and hydrated and underwent antigen retrieval, but frozen sections did not require this processing. The sections were treated with 5% bovine serum albumin and sealed at room temperature for 30 min. Then, diluted primary antibody was added to each section and incubated overnight in a wet box at 4 °C. After the excess primary antibody was washed away, the diluted fluorescent secondary antibody was added to the brain slice and incubated at 20–37 °C for 1 h in the dark. 4ʹ,6-diamino-2-phenylindole (DAPI) staining was performed for 5–10 min to label the nuclei. The sections were observed with an Olympus Automatic Scanning System SV120. The following primary antibodies were used: rabbit anti-IBA1 (NO. 019-19741, Wako), rabbit anti-iNOS (18985-1-AP, Proteintech), mouse anti-Arg-1 (66129-1-Ig, Proteintech), mouse anti-IBA1 (GB12105, Servicebio), and mouse anti-NLRP3 (AG-20B-0014-C100, Adipogen).

### Immunohistochemistry

Immunohistochemical staining was performed on paraffin-embedded sections. After the sections were dewaxed and rehydrated, 3% hydrogen peroxide solution was used to inactivate endogenous peroxidase. The next steps were the same as those for immunofluorescence staining until HRP-labeled secondary antibodies were administered, followed by the diaminobenzidine reaction. Nuclei were stained with hematoxylin as needed. For immunohistochemical staining, the primary antibodies used were as follows: rabbit anti-TH (25859-1-AP, Proteintech), rabbit anti-IBA1 (NO. 019-19741, Wako), and mouse anti-GFAP (60190-1-Ig, Proteintech).

### Statistical analysis

All data are presented as the means ± SEM and were analyzed using Prism 9 (GraphPad Software). Two-way ANOVA was used to analyze the differences in clock genes between the control group and MPTP group at different time points. Other data were analyzed using Student’s *t* test or one-way ANOVA with Dunnett’s post hoc test. A value of *p* < 0.05 was considered statistically significant throughout the study.

## Results

### Aberrant diurnal Rev-erbα rhythms in the SN of MPTP-induced PD mice

The mRNA levels of Rev-erbα in the SN were examined at different times within 24 h from the control and MPTP groups (Fig. 1A). As shown in control mice, Rev-erbα showed diurnal changes throughout the day, with the highest expression at ZT6 and the lowest expression at ZT18 (Fig. 1B). Contrarily, the diurnal oscillation of Rev-erbα in MPTP group disappeared (Fig. 1B; *p* < 0.05). Furthermore, the mean expression level of Rev-erbα in the MPTP group was significantly decreased (Fig. 1C; *p* < 0.001). To further verify these findings, we examined changes in protein levels corresponding to Rev-erbα mRNA expression. Similar results were found and mRNA expression of Rev-erbα lost diurnal fluctuations (Fig. 1D, E; *p* < 0.01). In addition, we detected the mRNA level changes of core circadian clock molecule Bmal1 and Per2 in the SN within 24 h, and the amplitude of Bmal1 and Per2 were also slightly attenuated (Additional file 1: Fig. S1A and C; both *p* < 0.05).

### Diurnal changes in microglia-mediated inflammatory cytokines expression in the SN of MPTP-induced PD mice

**Fig. 2:**
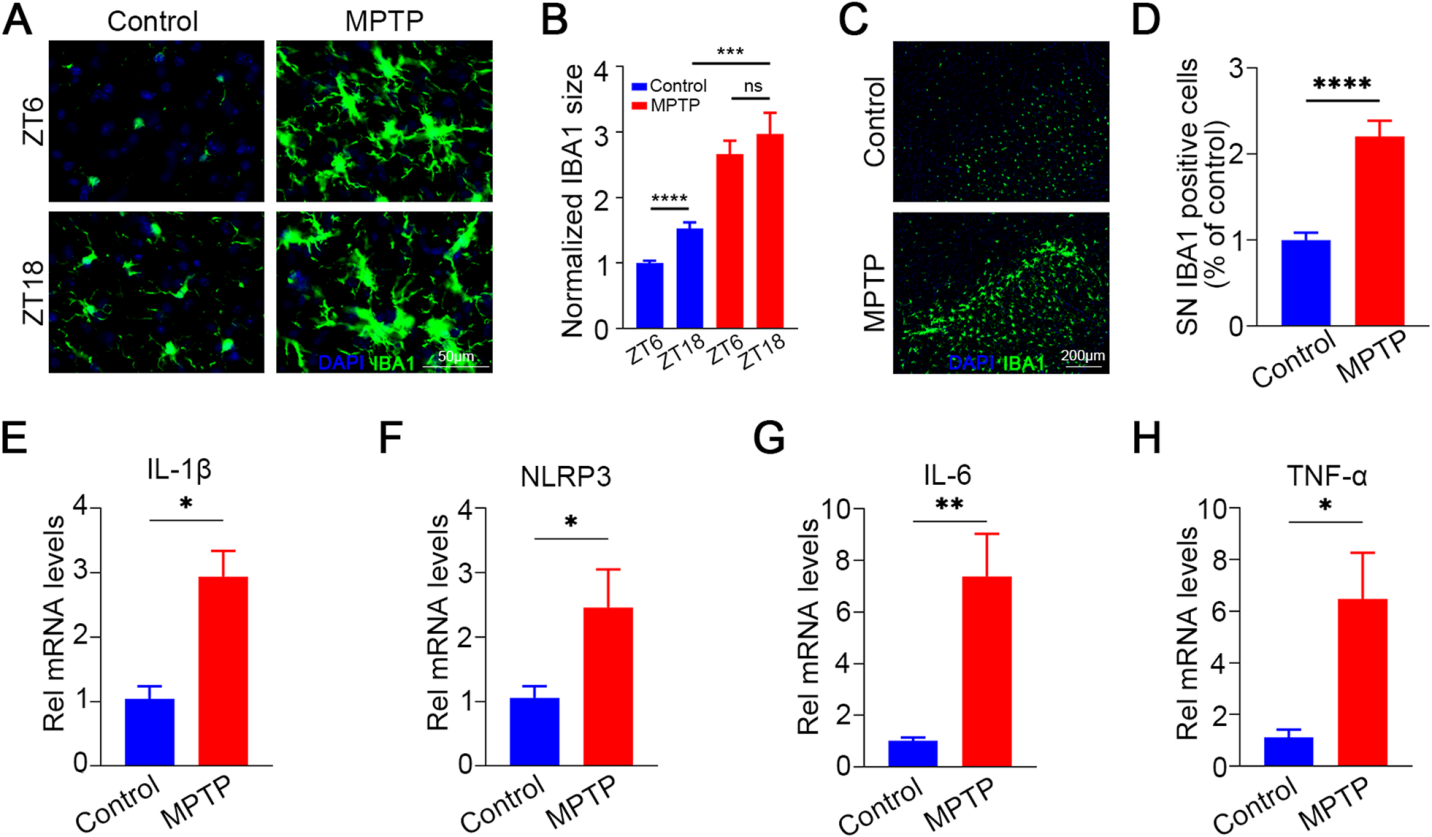
Diurnal changes in microglia and the inflammatory-related genes in SN of MPTP-induced Mice. **A** Representative images of IBA1 staining in the SN of control and MPTP mouse with IBA1 size quantification (**B**). Scale bar, 50 μm. Mice were killed at ZT6 and ZT18. **C** Iba1 staining in the control and MPTP group with Iba1 number quantification (**D**). Scale bar, 200 μm. **E–H** RT-PCR analysis for microglial inflammatory genes from SN of Control and MPTP mice. *n* = 3–4 for each group. Data were presented as mean ± SEM. (*p < 0.05, **p < 0.01, or ****p < 0.0001 by two-tailed *t* test)

The microglia-mediated neuroinflammation plays a vital role in PD pathogenesis [27]. Therefore, we examined morphological changes in microglia at different times to determine the relationship between Rev-erbα and microglial activation in the MPTP model. We first performed Iba1 immunofluorescent stain in SN at ZT6 and ZT18 time, which indicated the highest and lowest expression of Rev-erbα, respectively. Compared to control group, the diurnal change in the SN was abrogated in MPTP mice, as microglial size was significantly increased at ZT6 and ZT18, with no difference in the two time points (Fig. 2A, B). In addition, the number of microglia in the SN in the MPTP group was also significantly increased (Fig. 2C, D). Next, we used qRT-PCR to examine microglia-associated inflammatory cytokines, including IL-1β, NLRP3, IL-6 and TNF-α (Fig. 2E–H). The results showed that the average mRNA expression levels of these genes in the SN of MPTP mice were significantly higher than those in control group, indicating an activation of microglia-mediated neuroinflammation in MPTP mice.

Thus, we hypothesized that the loss of daily Rev-erbα fluctuations was the main factor contributing to the development of a proinflammatory microglial phenotype in MPTP mice, thereby mediating PD pathologic progression. Therefore, we further explored the effect and mechanism of Rev-erbα on microglia activation in BV2 cell, a mouse microglia cell line.

### Rev-erbα inhibited microglial activation induced by MPP^+^ and αsyn PFF

**Fig. 3:**
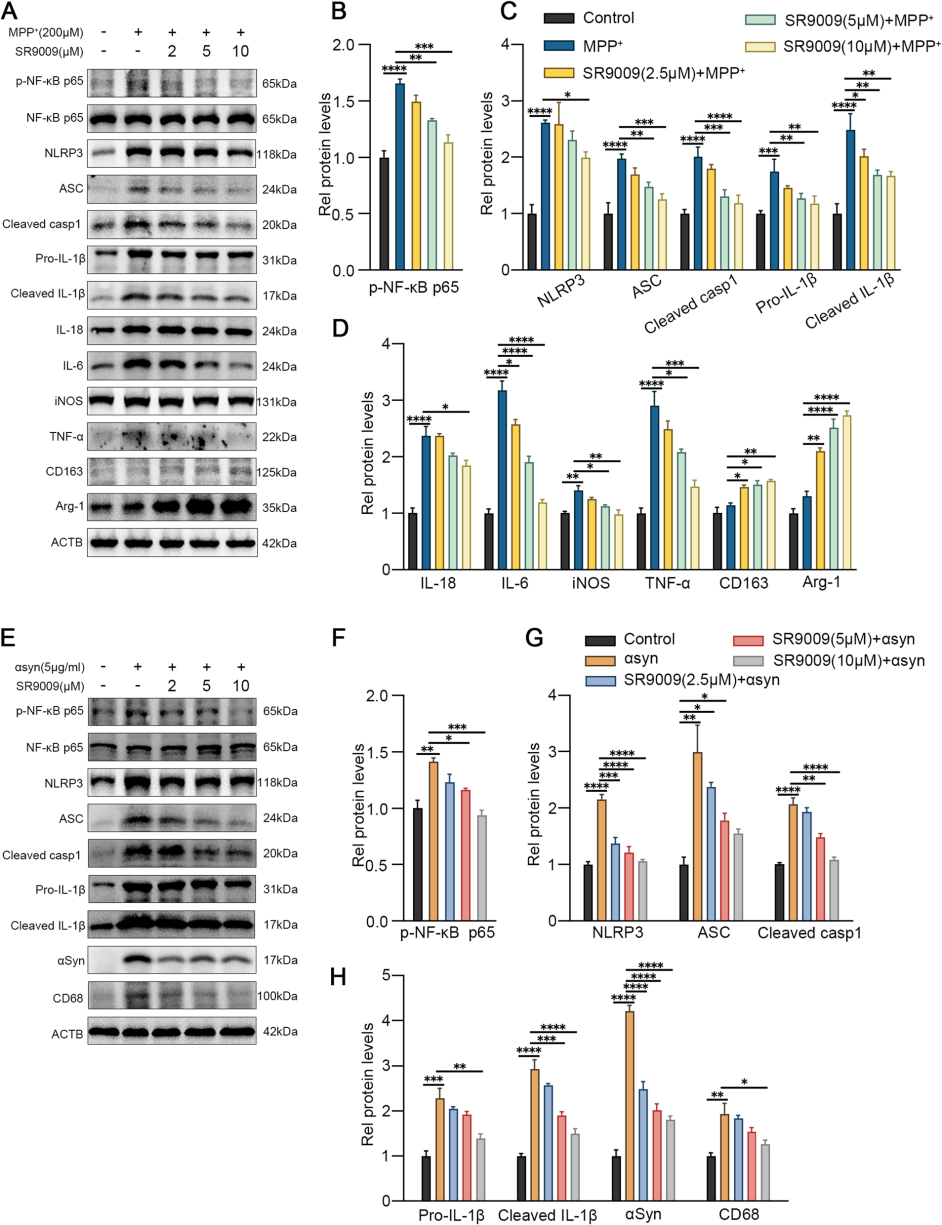
Activation of Rev-erbα inhibited microglial activation induced by MPP^+^ and αsyn PFF. The representative western blot bands (**A**) and the statistical graph (**B**–**D**) of p-NF-κB p65, NLRP3, ASC, cleaved caspase-1, IL-1β, IL-18, IL-6, TNF-a, iNOS, Arg-1 and CD163 protein expressions. BV2 cells were pretreated with SR9009 (2 μM, 5 μM, 10 μM) for 1 h, then incubated with MPP^+^ for 24 h. The representative western blot bands (**E**) and the statistical graph (**F–H**) of p-NF-κB p65, NLRP3, ASC, cleaved caspase-1, IL-1β, CD68 and αsyn protein expressions. BV2 cells were pretreated with SR9009 (2 μM, 5 μM, 10 μM) for 1 h, then incubated with αsyn pre-formed-fibril for 6 h. The p-NF-κB p65 level was normalized to the total of NF-κB p65, and the rest protein levels were normalized to β-actin. Data were presented as mean ± SEM (*n* = 3). (*p < 0.05, **p < 0.01, ***p < 0.001, **** or *p* < 0.0001 by One-way ANOVA test)

BV2 cells were pretreated with different concentrations of Rev-erbα agonist SR9009 (2 uM, 5 uM, 10 uM) for 1 h, and then treated with MPP^+^. Compared with control group, western blot showed that treatment with MPP^+^ significantly activated NF-κB and NLRP3 inflammasome in BV2 cells and promoted the expression of proinflammatory factors such as iNOS, IL-1β, IL-6 and TNF-α (Fig. 3A–D). However, these inflammatory responses were dose-dependently suppressed by SR9009, with increased expression of the anti-inflammatory cytokines Arg-1 and CD163 (Fig. 3A–D), indicating that SR9009 promoted MPP^+^-induced microglial transition from M1 to M2 type. Furthermore, we assessed the effects of SR9009 on the inflammatory response induced by αsyn PFF (Fig. 3E). Consistently, SR9009 effectively inhibited αsyn PFF-induced activation of NF-κB and NLRP3 inflammasome pathways (Fig. 3F–H). Furthermore, SR9009 reduced the deposition of αsyn PFF in BV2 cells (Fig. 3H). We further examined the phagocytosis of microglia, which was increased upon addition of αsyn PFF but decreased upon administration of SR9009 (Fig. 3H), suggesting that Rev-erbα may promote αsyn clearance by regulating microglial phagocytosis.

### Rev-erbα regulates microglial activation via NF-κB and NLRP3 inflammasome pathway

**Fig. 4:**
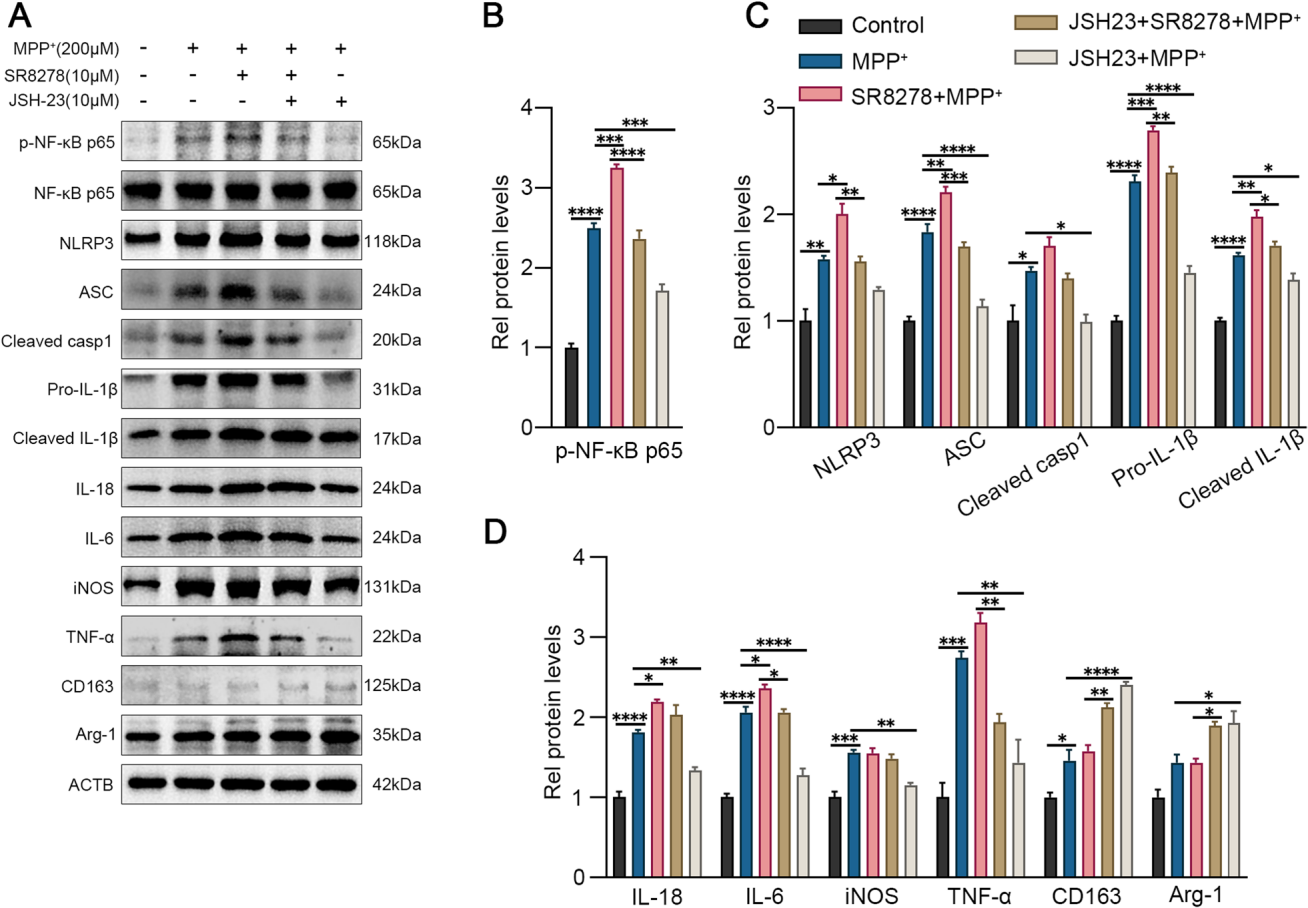
Rev-erbα regulates microglial activation by NF-κB inflammasome pathway. The representative western blot bands (**A**) and the statistical graph (**B**–**D**) of p-NF-κB p65, NLRP3, ASC, cleaved caspase-1, IL-1β, IL-18, IL-6, TNF-a, iNOS, Arg-1 and CD163 protein expressions. BV2 cells were pretreated with JSH-23 (10 μM) or SR8278 (10 μM) for 1 h, then incubated with MPP^+^ for 24 h. The p-NF-κB p65 level was normalized to the total of NF-κB p65, and the rest protein levels were normalized to β-actin. Data were presented as mean ± SEM (n = 3). (*p < 0.05, **p < 0.01, ***p < 0.001, **** or *p* < 0.0001 by One-way ANOVA test)

We further elucidated the potential mechanism by which Rev-erba regulates microglial activation through SR8278 (Rev-erba inhibitor) and JSH-23 (NF-κB inhibitor). As shown in Fig. 4A, p-NF-κB p65, NLRP3, ASC, IL-18, cleaved caspase-1 and IL-1β, as well as other inflammatory cytokines iNOS, IL-6, and TNF-α, were increased in the MPP^+^ group compared with the control group (Fig. 4B–D, all *p* < 0.05). In addition, the activation of microglia was more pronounced after SR8278 pretreatment while the inflammatory cytokines decreased and anti-inflammatory factors increased after JSH-23 pretreatment, suggesting that Rev-erba may alleviate MPP^+^-induced microglial activation and promote the transformation of microglia from M1 type to M2 type through the NF-κB pathway.

**Fig. 5:**
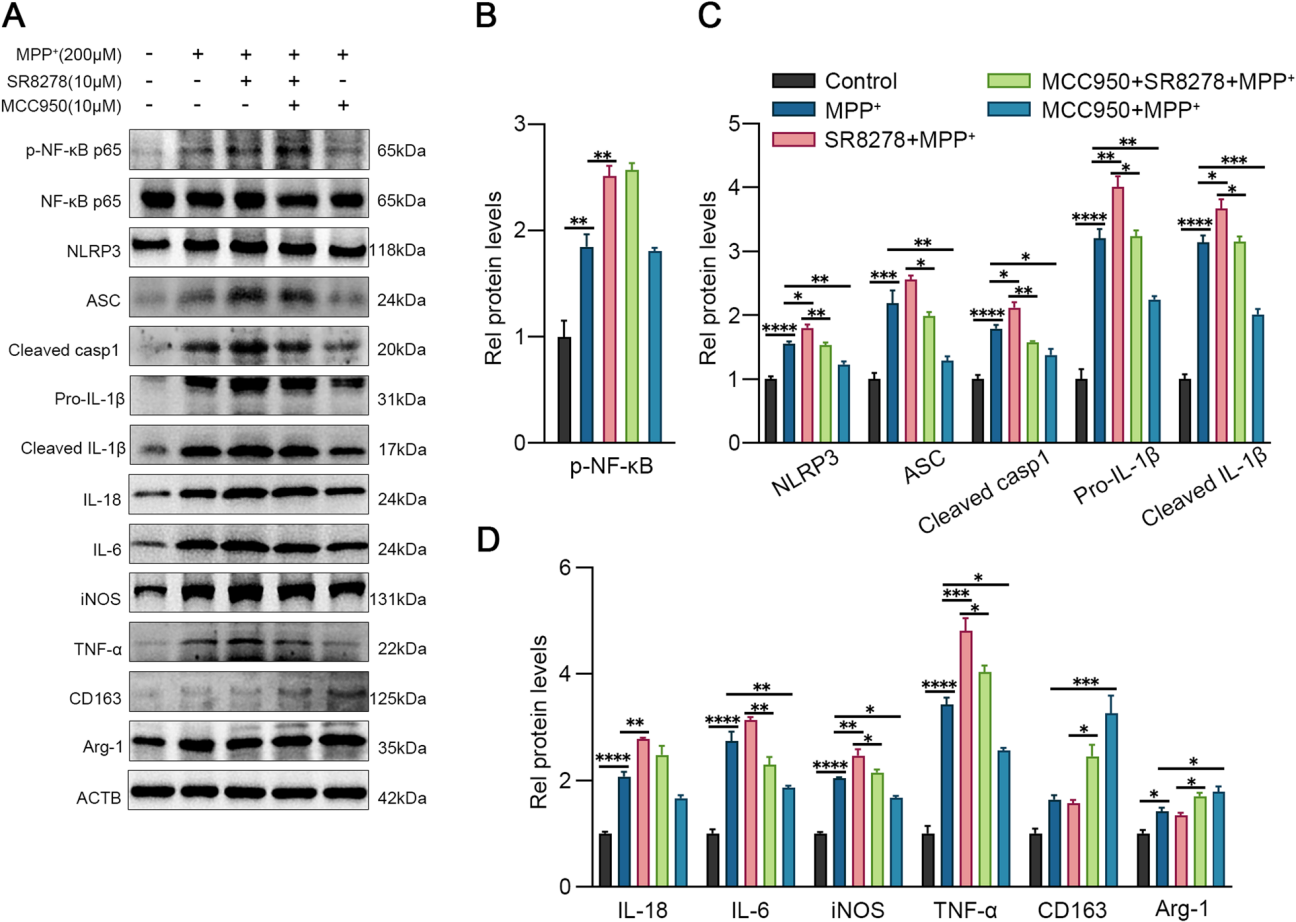
Rev-erbα regulates microglial activation by NLRP3 inflammasome pathway. The representative western blot bands (**A**) and the statistical graph (**B**–**D**) of p-NF-κB p65, NLRP3, ASC, cleaved caspase-1, IL-1β, IL-18, IL-6, TNF-a, iNOS, Arg-1 and CD163 protein expressions. BV2 cells were pretreated with MCC950 (10 μM) or SR8278 (10 μM) for 1 h, then incubated with MPP^+^ for 24 h. The p-NF-κB p65 level was normalized to the total of NF-κB p65, and the rest protein levels were normalized to β-actin. Data were presented as mean ± SEM (*n* = 3). (*p < 0.05, **p < 0.01, ***p < 0.001, **** or *p* < 0.0001 by One-way ANOVA test)

To further investigate whether Rev-erba modulates the inflammatory phenotype of microglia via the NLRP3 inflammasome, we pretreated BV2 cells with MCC950, a classic NLRP3 inflammasome inhibitor. Western blot results (Fig. 5A) showed that compared with the MPP^+^ group, MCC950 administration significantly inhibited the activation of NLRP3 inflammasome, decreased the expression of proinflammatory factors (iNOS, IL-6 and TNF-a), and increased the expression of anti-inflammatory factors such as Arg-1 and CD163 (Fig. 5C, D, both *p* < 0.05), but the expression of p-NF-κB p65 was not affected (Fig. 5B, *p* > 0.05). In comparison with the SR8278 + MPP^+^ group, the MCC950 + SR8278 + MPP^+^ group also showed a similar trend, further suggesting that the NLRP3 inflammasome pathway plays a key role in the regulation of Rev-erba on neuroinflammation.

### SR9009 improves motor function in MPTP-induced PD mice

**Fig. 6:**
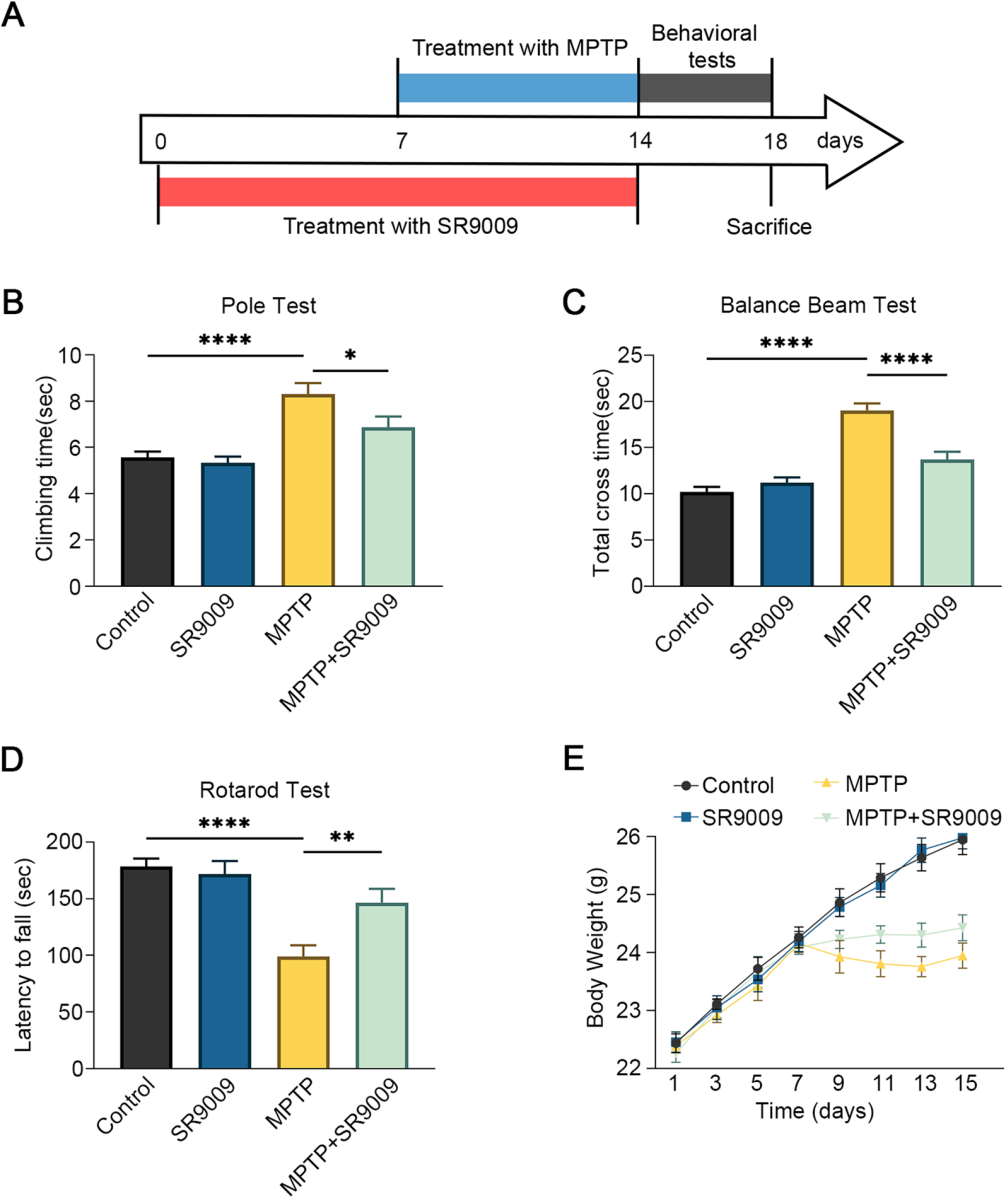
SR9009 ameliorates behavioral impairments in MPTP-induced Mice. **A** Schematic representation of SR9009 intervention therapy. SR9009 was injected intraperitoneally (100 mg/kg/day) for 7 consecutive days prior to MPTP administration and was continued along with MPTP treatment. Behavioral tests began the day after completing 14 days of SR9009 treatment, and mice were killed after behavioral test (day 18). **B** Time spent in climbing of the pole test. **C** The time taken to cross the balance beam. **D** Latency to fall of the rotarod test. **E** Body-weight changes over time. *n* = 10 for each group. Data were presented as mean ± SEM. (*p < 0.05, **p < 0.01, or ****p < 0.0001 by One-way ANOVA test)

Based on the above studies, our next step was to explore the regulatory effects of SR9009 on motor function, dopaminergic neuron loss and microglia-mediated neuroinflammation in the MPTP model (Fig. 6A). Behavioral tests were performed including pole test, rotarod test and balance beam test. In comparison with that of the control group, the pole test indicated a significantly prolonged time in MPTP group (*p* < 0.0001, Fig. 6B). Similar results were found in the balance beam test (*p* < 0.0001, Fig. 6C). It should be noted that Rev-erbα agonist SR9009 partially reversed these effects (both *p* < 0.05, Fig. 6B, C). Similarly, SR9009 partially reversed MPTP-induced latency shortening on the rotational axis (*p* < 0.01, Fig. 6D). In addition, SR9009 was texted to be no influence on body weights of mice (Fig. 6E). These data indicate that SR9009 improves motor function of MPTP-induced mice.

### SR9009 protects against nigrostriatal dopaminergic degeneration in the SN of MPTP-induced mice

**Fig. 7:**
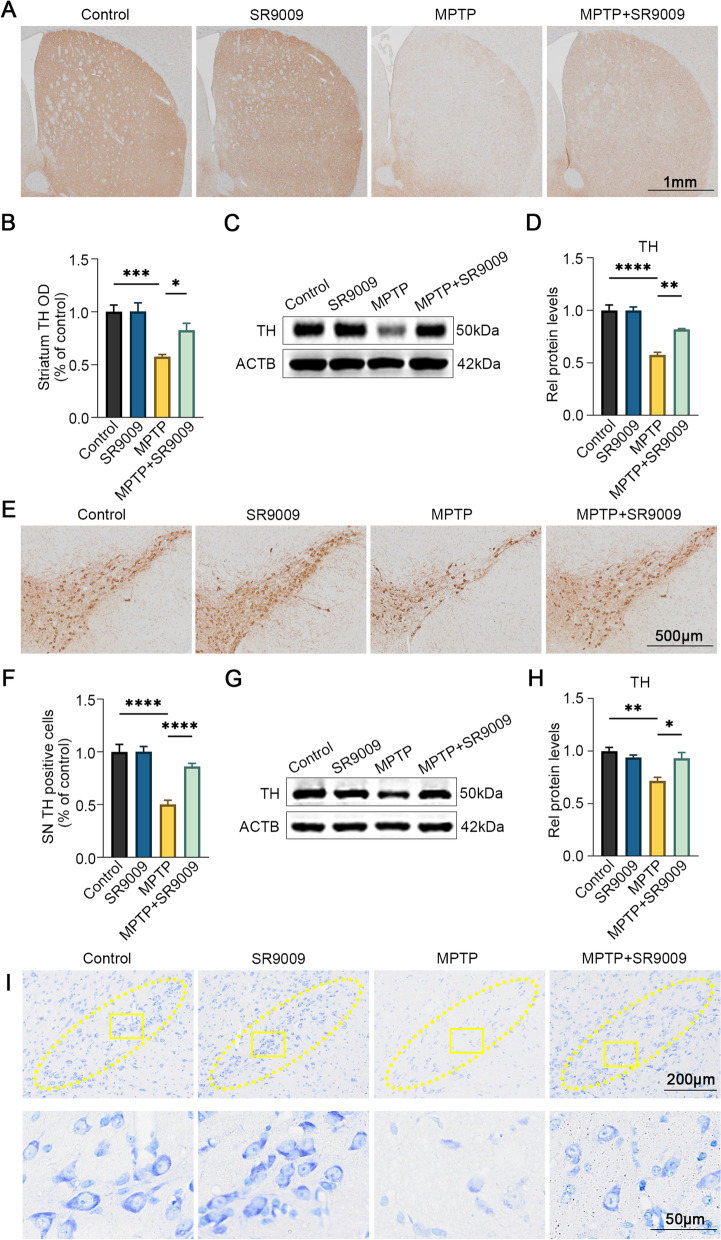
SR9009 protects against dopaminergic neurons degeneration in MPTP-induced mice. The representative immunohistochemical staining of TH (**A**) and the OD of TH staining (**B**) in the striatum. Scale bar, 1 mm. Representative western blot bands (**C**) and the statistical graph (**D**) of TH in the striatum. The representative immunohistochemical staining of TH (**E**) and the number of TH positive neurons (**F**) in the SN. Scale bar, 500 μm. Representative western blot bands (**G**) and the statistical graph (**H**) of TH in the SN. **I** The representative Nissl staining for neurons in the SN. Scale bars, 200 μm for the top row and 50 μm for the bottom row. The TH protein level was normalized to β-actin. *n* = 3–4 for each group. Data were presented as mean ± SEM. (*p < 0.05, **p < 0.01, or ***p < 0.001 by one-way ANOVA test)

To assess whether SR9009 affects MPTP-dependent neural loss, we evaluated nigrostriatal dopaminergic degeneration. Immunohistochemical analysis and western blotting showed that dopaminergic neurons in the striatum (all *p* < 0.05, Fig. 7A–D) and SN (all *p* < 0.05, Fig. 7E–H) in the MPTP group were severely damaged in comparison with those in the control group, but this effect was largely reversed by SR9009. The neuroprotective effect of SR9009 was further confirmed by Nissl staining (Fig. 7I), which demonstrated partial preservation of dopaminergic neurons in the SN in the MPTP + SR9009 group. These results suggest that SR9009 partially prevents MPTP-induced dopaminergic loss.

### SR9009 ameliorates microgliosis and astrocytosis in the SN of MPTP-induced mice

**Fig. 8:**
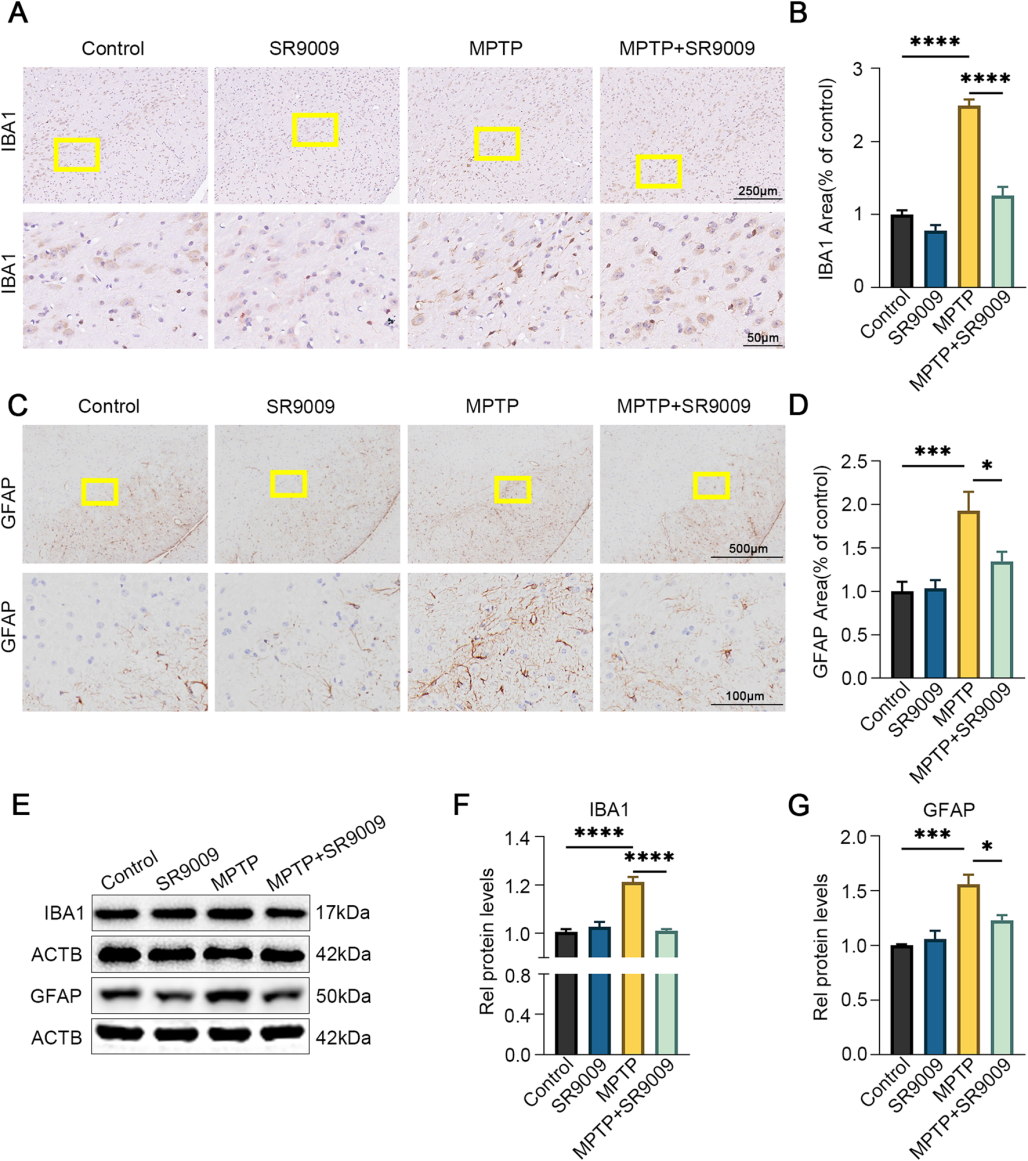
SR9009 inhibits microgliosis and astrocytosis in the SN of MPTP-induced mice. The representative immunohistochemistry staining (**A**) and the statistical graph (**B**) of IBA1 in the SN. Scale bars, 250 μm for the top row and 50 μm for the bottom row. The representative immunohistochemistry staining (**C**) and the statistical graph (**D**) of GFAP in the SN. Scale bars, 500 μm for the top row and 100 μm for the bottom row. Representative western blot bands (**E**) and the statistical graph (**F**, **G**) of IBA1 and GFAP in the SN. The protein levels were normalized to β-actin. *n* = 3–4 for each group. Data were presented as mean ± SEM. (*p < 0.05, **p < 0.01, or ***p < 0.001 by one-way ANOVA test)

Glial activation is an important pathological marker of PD neuroinflammation [28, 29]. Therefore, the effect of SR9009 on the activation of microglia and astrocytes in the SN was examined. Immunohistochemical staining showed that compared with that in the control group, the number of microglia and astrocytes in the SN in MPTP group was significantly increased, indicating an activated state (Fig. 8A–D, the control group vs. the MPTP group, both *p* < 0.05). Moreover, we found that glial activation was partly abolished in the SN in the MPTP + SR9009 group (Fig. 8A–D, the MPTP + SR9009 group vs. the MPTP group, both *p* < 0.05). In comparison with the control, MPTP strongly increased IBA1 and GFAP expression (Fig. 8E–G, both *p* < 0.05). Compared with those in the MPTP group, the protein levels of IBA1 and GFAP in the SN in the MPTP + SR9009 group were decreased (Fig. 8E–G, both *p* < 0.05). These results indicate that SR9009 partially ameliorates MPTP-induced glial cell overactivation in the SN of MPTP-induced mice.

### SR9009 ameliorates microgliosis and astrocytosis in the striatum of MPTP-induced mice

**Fig. 9:**
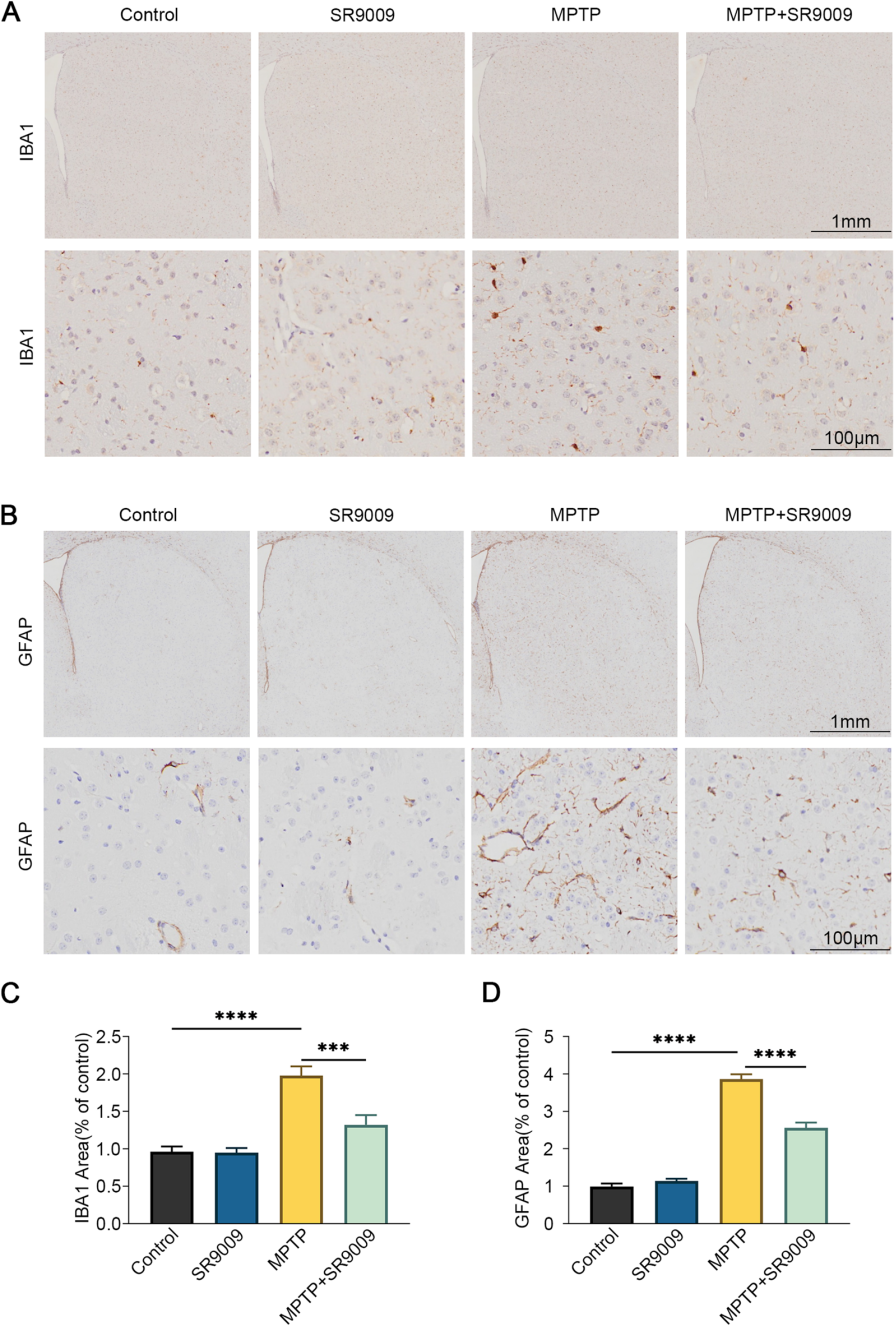
SR9009 inhibits microgliosis and astrocytosis in striatum of MPTP-induced mice. **A** The representative immunohistochemistry staining of IBA1 in the striatum. Scale bars, 1 mm for the top row and 100 μm for the bottom row. **B** The representative immunohistochemistry staining of GFAP in the striatum. Scale bars, 1 mm for the top row and 100 μm for the bottom row. **C**, **D** The statistical graph of IBA1 and GFAP in the striatum. (*p < 0.05, **p < 0.01, or ***p < 0.001 by One-way ANOVA test)

In addition to SN area, the striatum is also an important part of the dopaminergic system. Immunohistochemical staining showed that compared with that in the control group, microglia and astrocytes in the striatum in the MPTP group was significantly activated (Fig. 9A–D, the control group vs. the MPTP group, both *p* < 0.05). However, the glial activation was partly abolished in the striatum in the MPTP + SR9009 group (Fig. 9A–D, the MPTP + SR9009 group vs. the MPTP group, both *p* < 0.05). These results further indicate that SR9009 can partially ameliorate MPTP-induced glial cell overactivation in the nigro striatum of MPTP-induced mice.

### SR9009 reverses the phenotypic polarization of microglia in the SN of MPTP-induced mice

**Fig. 10:**
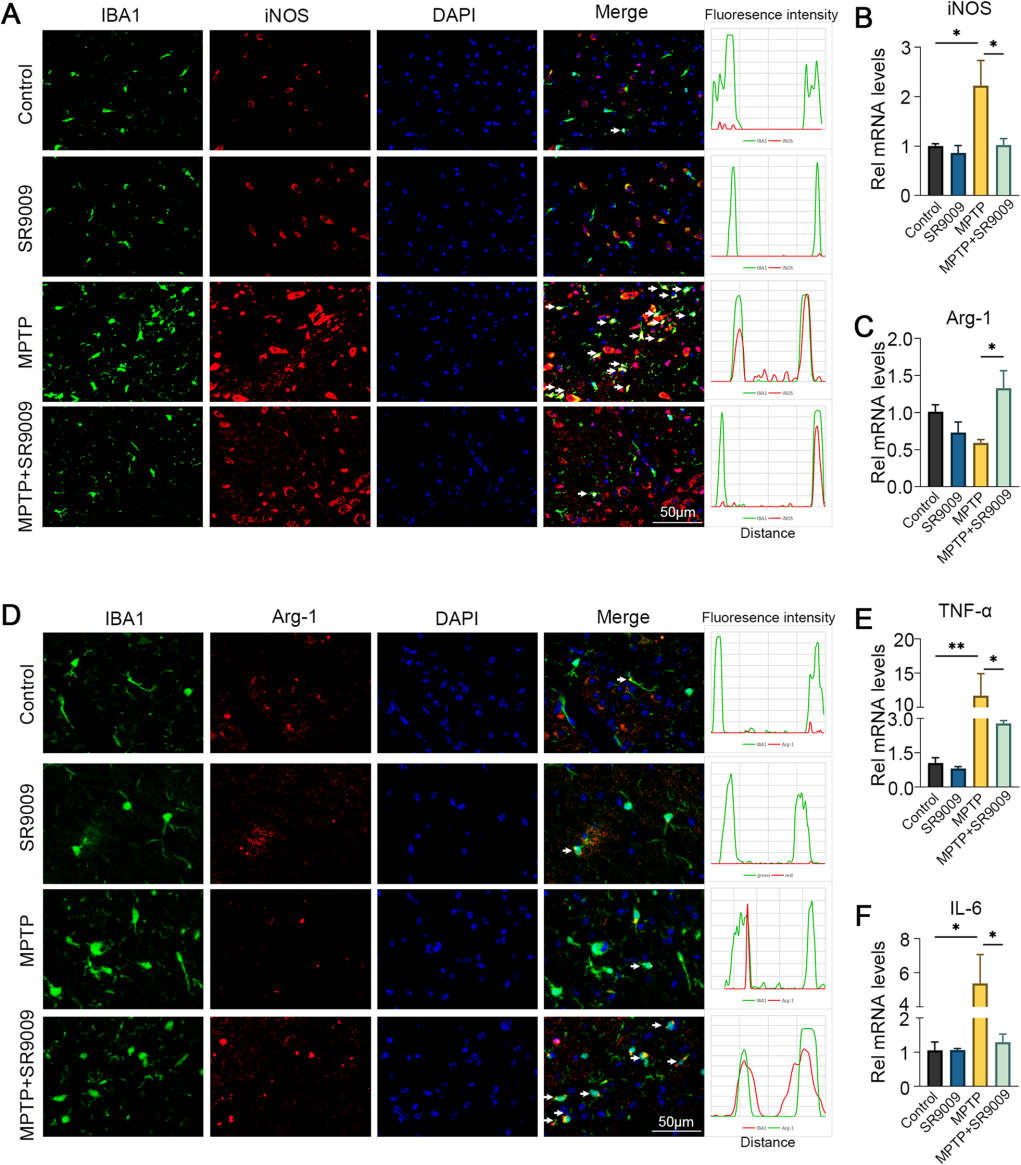
SR9009 reversed the phenotypic polarization of microglia in the SN of MPTP-induced mice. **A** Representative double-immunofluorescent staining of IBA1 (green) and iNOS (red) in the SN. Scale bar, 50 μm. **D** Representative double-immunofluorescent staining of IBA1 (green) and iNOS (Arg-1) in the SN. Scale bar, 50 μm. The real-time PCR results of iNOS (**B**), Arg-1 (**C**), TNF-α (**E**) and IL-6 (**F**) in the SN. *n* = 3–4 for each group. Data were presented as mean ± SEM. (*p < 0.05, **p < 0.01, or ***p < 0.001 by One-way ANOVA test)

Next, we sought to determine whether SR9009 could modulate the phenotypic polarization of microglia. To examine phenotypic polarization, immunofluorescence stain was performed against iNOS (M1 phenotypic marker) and Arg-1 (M2 phenotypic marker). INOS^+^ IBA1^+^ cells and Arg^+^ IBA1^+^ cells were observed, as shown in Fig. 10A and D. The amount of iNOS co-localized with IBA1 in the MPTP + SR9009 group was less than that in the MPTP group (Fig. 10A), while the amount of Arg-1 co-localized with IBA1 in the MPTP + SR9009 group was higher than that in MPTP group (Fig. 10D). Similar to these results, the mRNA levels of iNOS and Arg-1 showed the same trend (Fig. 10B, C, both *p* < 0.05). In addition, the PCR results showed that SR9009 significantly reduced the microglia-associated proinflammatory factors TNF-α and IL-6 in MPTP group (Fig. 10E, F, both *p* < 0.05). These results demonstrated that SR9009 promoted transformation from the M1 phenotype to the M2 phenotype in MPTP-induced mice.

### SR9009 partially inhibits NLRP3 inflammasome activation in the SN of MPTP-induced mice

**Fig. 11:**
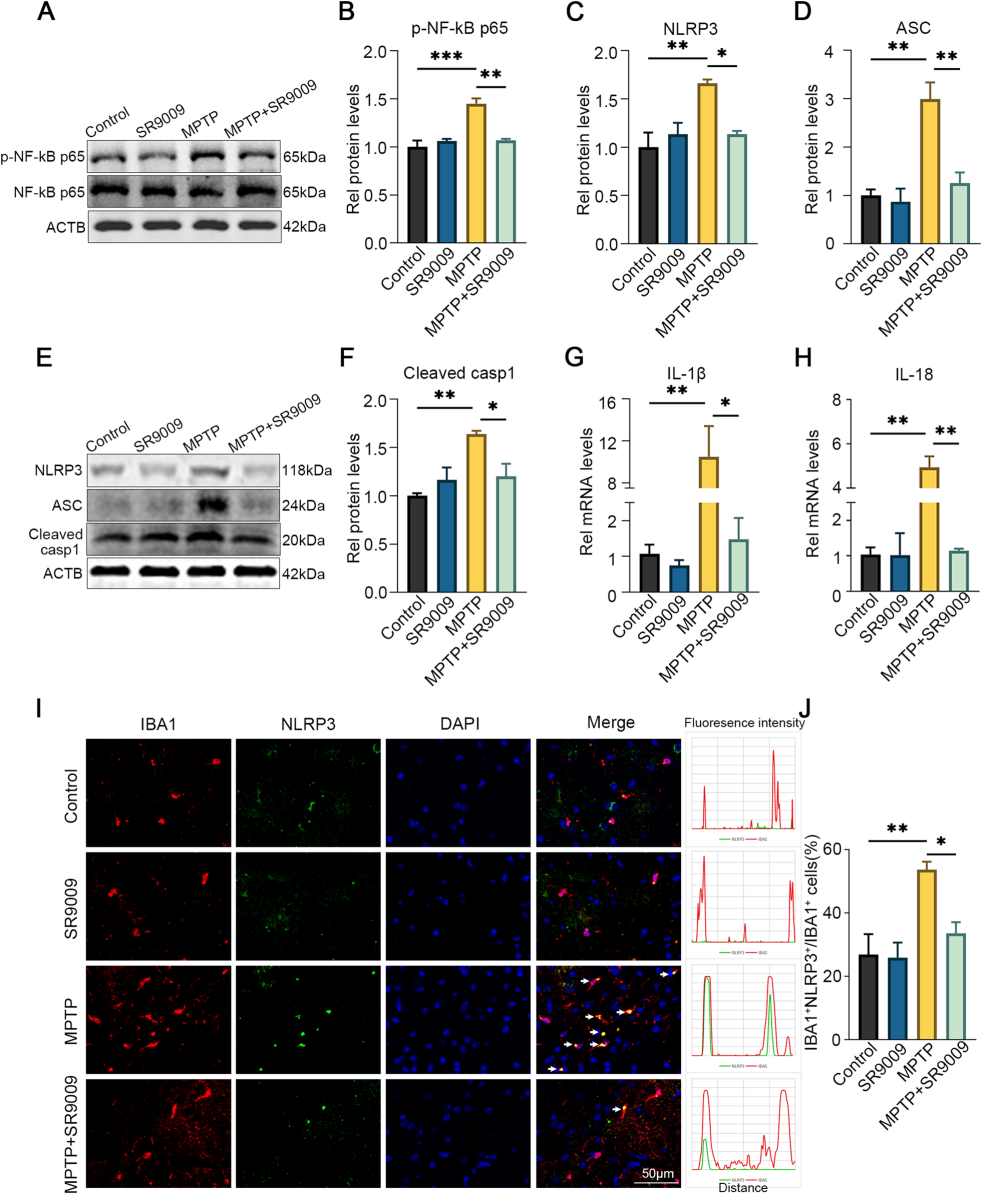
SR9009 suppresses NLRP3 inflammasome activation in the SN of MPTP-induced mice. The representative western blot bands (**A**) and the statistical graph (**B**) of p-NF-κB p65 and NF-κB p65 in the SN. The representative western blot bands (**E**) and the statistical graph (**C**–**D**, **F**) of NLRP3, ASC and cleaved-caspase-1 in the SN. The real-time PCR results of IL-1β (**G**) and IL-18 (**H**) in the SN. **I** Representative double-immunofluorescent staining of IBA1 (red) and NLRP3 (green) in the SN and the statistical graph (**J**) of IBA1^+^ NLRP3^+^/IBA1^+^ cells. Scale bar, 50 μm. *n* = 3–4 for each group. The p-NF-κB p65 level was normalized to the total of NF-κB p65, and the rest protein levels were normalized to β-actin. Data were presented as mean ± SEM. (*p < 0.05, **p < 0.01, or ***p < 0.001 by One-way ANOVA test)

Since there is increasing evidence that the NLRP3 inflammasome is involved in PD progression [12, 30], we next investigated the effect of SR9009 on NLRP3 inflammasome activation in the MPTP model. The effects of SR9009 on NF-κB, which is an essential priming effector for inflammasome activation and the core components of the NLRP3 inflammasome, were examined by western blotting (Fig. 11A–F). In comparison with that in control mice, the expression of p-NF-κB p65, NLRP3, cleaved caspase-1 and ASC in the SN of MPTP-induced mice was significantly increased (all *p* < 0.05), suggesting the activation of the NLRP3 inflammasome. However, SR9009 effectively reversed the increase in p-NF-κB p65, NLRP3, ASC and cleaved caspase-1 in the SN (MPTP + SR9009 group vs. MPTP group, all *p* < 0.05). In addition, the PCR data also showed that SR9009 effectively reduced the production of the inflammatory cytokines IL-1β and IL-18 in the MPTP-induced mice (Fig. 11G, H, the MPTP + SR9009 group vs. the MPTP group, all *p* < 0.05). Immunofluorescence colocalization analysis also showed that NLRP3 was mainly expressed in microglia in the MPTP group, and this effect was partially reversed in the MPTP + SR9009 group (Fig. 11I, J). These data suggest that MPTP-induced NLRP3 inflammasome activation in the SN can be partially blocked by SR9009.

## Discussion

**Fig. 12:**
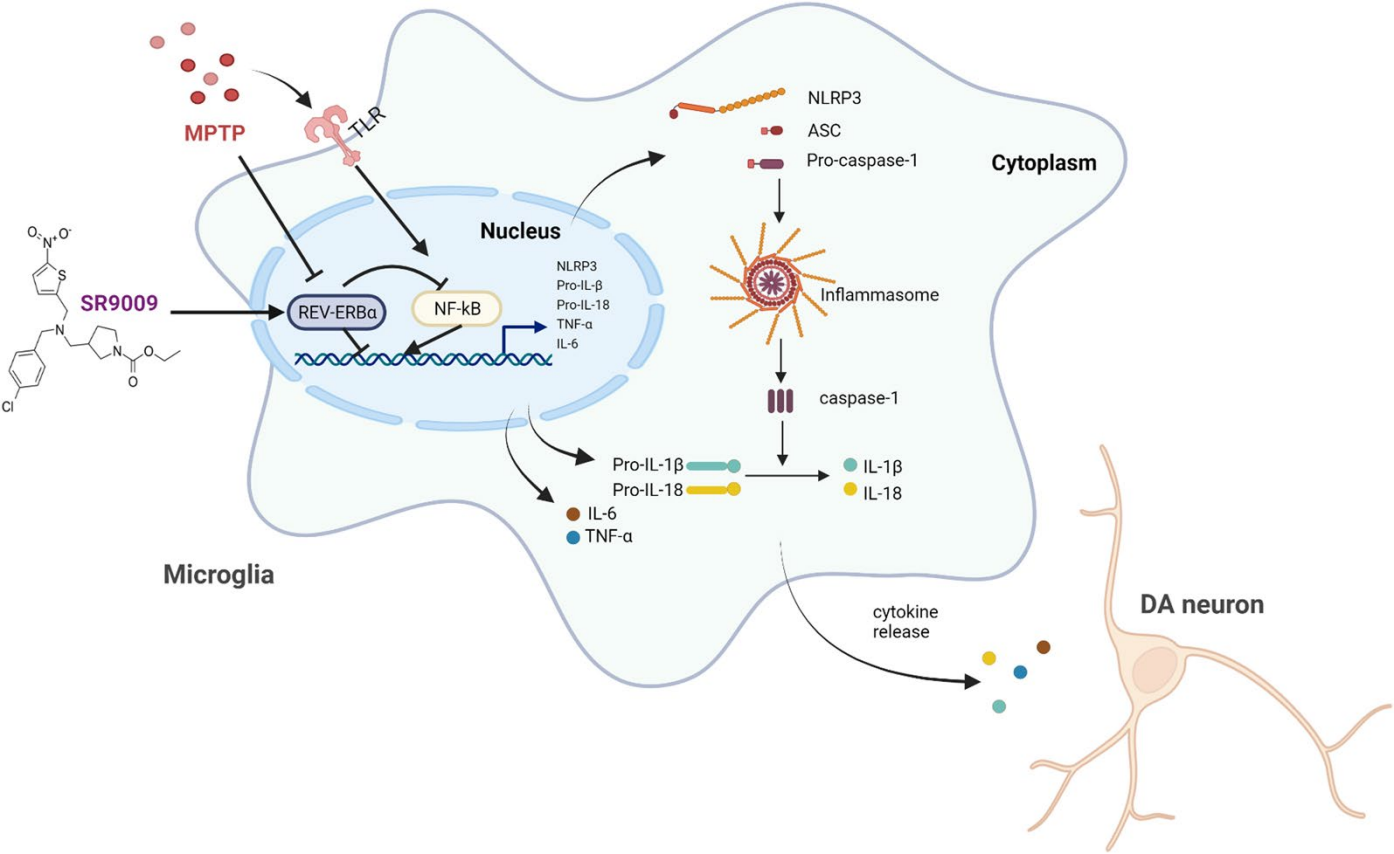
The mechanism underlying Rev-erbα /NF-κB/NLRP3 axis in MPTP-induced microglia neuroinflammation and dopaminergic impairment. MPTP upregulates the phosphorylation of NF-κB, then activates the NLRP3 inflammasome and promotes the release of inflammatory cytokines. These result in glial proliferation and microglial polarization, ultimately induced dopaminergic impairment. In addition, Rev-erbα-specific small molecule agonist SR9009 alleviates neuroinflammation and improves neurologic outcomes in the MPTP model

In this study, we showed for the first time that the normal daily fluctuations of Rev-erbα were absent in subacute MPTP-induced PD model. In addition, the diurnal variations in microglial immunoreactivity in the SN disappeared, suggesting that decreased expression of Rev-erbα may be responsible for microglial activation and elevated neuroinflammation. Then we demonstrated that Rev-erbα can regulate microglial activation and polarization through the NF-κB and NLRP3 inflammasome pathways in vitro. Moreover, our results further revealed that activation of Rev-erbα by the small molecule agonist SR9009 could improve motor function, ameliorate dopaminergic neurons loss, inhibit gliosis and microglial polarization by regulating NLRP3 inflammasome activation in vivo. In summary, our results indicate that Rev-erbα is involved in the regulation of neuroinflammation in the pathological process of PD and is a potential new target for PD treatment (Fig. 12).

A great deal of researches indicates that circadian disturbance may not only be a concomitant symptom, but also a cause of neurodegenerative disease [31, 32]. Our previous study also confirmed that most PD patients were associated with disturbed sleep–wake cycles, indicating circadian rhythm disruption [33]. Rev-erbα, a known circadian modulator playing a pivotal role in the cyclic Bmal1 and CLOCK transcription [34, 35], has been implicated in a variety of physiological and pathophysiological processes, such as metabolism [36], cancers [37, 38], and inflammatory responses [39]. Moreover, Rev-erbα seems to be a double-edged sword in neurodegenerative disease, exacerbating amyloid-β (Aβ) deposition in Alzheimer’s disease while suppressing neuroinflammation in a model of epilepsy [40, 41], but its role in PD pathology is poorly understood. In the present study, we are the first to demonstrate that Rev-erbα is reduced and loses diurnal fluctuations in the SN in the MPTP model.

It is well known that microglial activation is an important part of the pathogenesis of PD [42]. Our previous studies have shown that microglia play a key role in the delivery of α-synuclein via exosomes [26], and activated microglia facilitate the transport of exosomal α-synuclein through Toll-like receptor 2 [43]. Microglia-mediated neuroinflammation in PD has been the focus of our attention [44]. In this study, we observed changes in microglial volume over time in the control group, which was significantly higher at ZT18 than at ZT6 (corresponding to the times of the lowest and highest Rev-erbα expression, respectively). However, this diurnal change was abrogated in the MPTP group, and microglial volume was significantly increased at ZT6 and ZT18. This finding is similar to that of a study showing that the deletion of Rev-erbα caused microglia to lose time-of-day changes and switch to a proinflammatory state [22], suggesting that Rev-erbα may mediate neuroinflammation in the MPTP model.

The NLRP3 inflammasome, an important component of inflammation, is a complex of multiple proteins, including NLRP3, ASC and caspase-1, which are expressed abundantly in microglia [17, 45]. When stimulated, the NLRP3 complex leads to caspase-1 activation, promotes the maturation and secretion of the proinflammatory cytokines IL-1β and IL-18, and induces the shift of microglia into an anti-inflammatory state [46], while inhibition the NLRP3 inflammasome suppresses microglial polarization to a proinflammatory state [47]. The link between Rev-erbα and microglial activation was investigated in BV2 cell. We found that activation of Rev-erbα could attenuate microglial activation and promote the transformation of microglia from proinflammatory M1 to anti-inflammatory M2 state through NF-κB and NLRP3 inflammasome pathway, while inhibition of Rev-erbα caused the opposite effect, which was consistent with other researches [23, 39, 48]. Notably, Rev-erbα not only inhibited MPP^+^ induced microglial activation, but also significantly attenuated αSyn PFF-induced NLRP3 inflammasome activation. More importantly, unlike previous studies suggesting that inhibition of Rev-erbα can reduce amyloid plaque in microglia [40], our study found that activation of Rev-erbα can significantly reduce αSyn deposition in BV2 cells. In addition, existing evidence shows extensive NLRP3 inflammasome activation in the SN of postmortem PD brains [11]. Neurotoxins such as MPTP, 6-OHDA, and αsyn PFF can induce the activation of the NLRP3 inflammasome [49–51], and inhibiting NLRP3 inflammasomes can effectively prevent dopaminergic neurodegeneration in PD models [51, 52]. Consistent with the above research, we detected the activation of the NLRP3 inflammasome in the SN in the MPTP model, while the administration of SR9009 effectively inhibited NLRP3 inflammasome activation and the release of the inflammatory factors IL-1β and IL-18. This effect is similar to the anti-inflammatory effect of SR9009 on colitis [48], hepatitis [53], pneumonia [54] and other inflammatory diseases [55], which further supports that the NLRP3 inflammasome is under the control of circadian rhythm, and Rev-erbα may play an anti-inflammatory and neuroprotective role by regulating the NLRP3 inflammasome in PD.

Based on previous experimental results, we first explored the effect of Rev-erbα agonist SR9009 on motor manifestations and typical PD pathology in vivo. Surprisingly, SR9009 not only significantly improved MPTP-induced dopaminergic neuron loss in the SN and striatum in mice but also reversed multiple motor dysfunctions induced by MPTP. Combined with the findings of other studies, chronic circadian disruption or genetic abrogation of Rev-erbα can exacerbate the deterioration of motor deficits and dopaminergic neuron damage in PD mice [7, 24]. These results indicate that abnormal reductions in Rev-erbα are an important risk factor for PD, and intervention with Rev-erbα may be a potential strategy for the treatment of neurodegenerative diseases.

In addition to microglia, astrocyte activation is also an important feature of PD neuroinflammation [7]. Our results confirmed that the small molecule agonist of Rev-erbα SR9009 effectively inhibited activation of microglia and astrocytes in the nigrostriatal system of MPTP-induced mice. After MPTP stimulation, SN microglia changed from a resting state to an activated state, and most of these cells transformed into the proinflammatory M1 phenotype. However, SR9009 effectively reversed this harmful change and converted microglia into the anti-inflammatory M2 phenotype. These results indicate that Rev-erbα is involved in regulating glial proliferation and microglia polarization in the MPTP model.

In this study, we found that in the MPTP model, the expression pattern of Rev-erbα was disordered and the average expression level decreased, but the mechanism was unclear. Some studies have shown that inflammatory challenges can cause significant changes in Rev-erbα protein stability and degradation [54]; thus, we hypothesized that the decline in Rev-erbα in the PD model may also be associated with MPTP-induced neuroinflammation. In summary, circadian disruption and neuroinflammation may play a synergistic role in the pathological progression of PD, and stabilizing circadian rhythm is not only helpful in alleviating patient symptoms but may even modify disease progression.

## Conclusions

In summary, abnormal expression of Rev-erbα is a key factor in regulating neuroinflammation in the SN in the MPTP model, and restoring stable circadian rhythm may be an effective strategy for delaying or even stopping the progression of neurodegenerative disease.

## Supplementary Information

**Additional file 1: Figure S1.** Aberrant diurnal Bmal1 and Per2 rhythm in the SN of MPTP-induced Mice. (A and C) The mRNA level of Bmal1 and Per2 in the SN was quantified using real-time PCR. (*p < 0.05, two-way ANOVA test, interaction between time and genotypes). (B and D) The average values of Bmal1 and Per2 over the course of the day were calculated. (two-way ANOVA test, interaction between genotypes). *n* = 3–5 for each time point. Data were presented as mean ± SEM.

## Data Availability

The data used during the current study are available from the corresponding author on reasonable request.

## Abbreviations

PD: Parkinson's disease
MPTP: 1-Methyl-4-phenyl-1,2,3, 6-tetrahydropyridyl
SN: Substantia nigra
SNpc: Substantia nigra pars compacta
CNS: Central nervous system
NLRP3: NLR Family Pyrin Domain Containing 3
APP-KI: Amyloid precursor protein knock-in
RT-PCR: Quantitative real-time PCR
ASC: Apoptosis-associated speck-like protein
TH: Tyrosine hydroxylase
HUST: Huazhong University of Science and Technology
IL-1β: Interleukin-1β
TNF-α: Tumor necrosis factor-α
