# Supplementary figures and images for: The circadian clock protein Rev-erbα provides neuroprotection and attenuates neuroinflammation against Parkinson’s disease via the microglial NLRP3 inflammasome
Source: J Neuroinflammation. 2022 Jun 6;19:133. doi: 10.1186/s12974-022-02494-y (PMC9169406; doi:10.1186/s12974-022-02494-y)

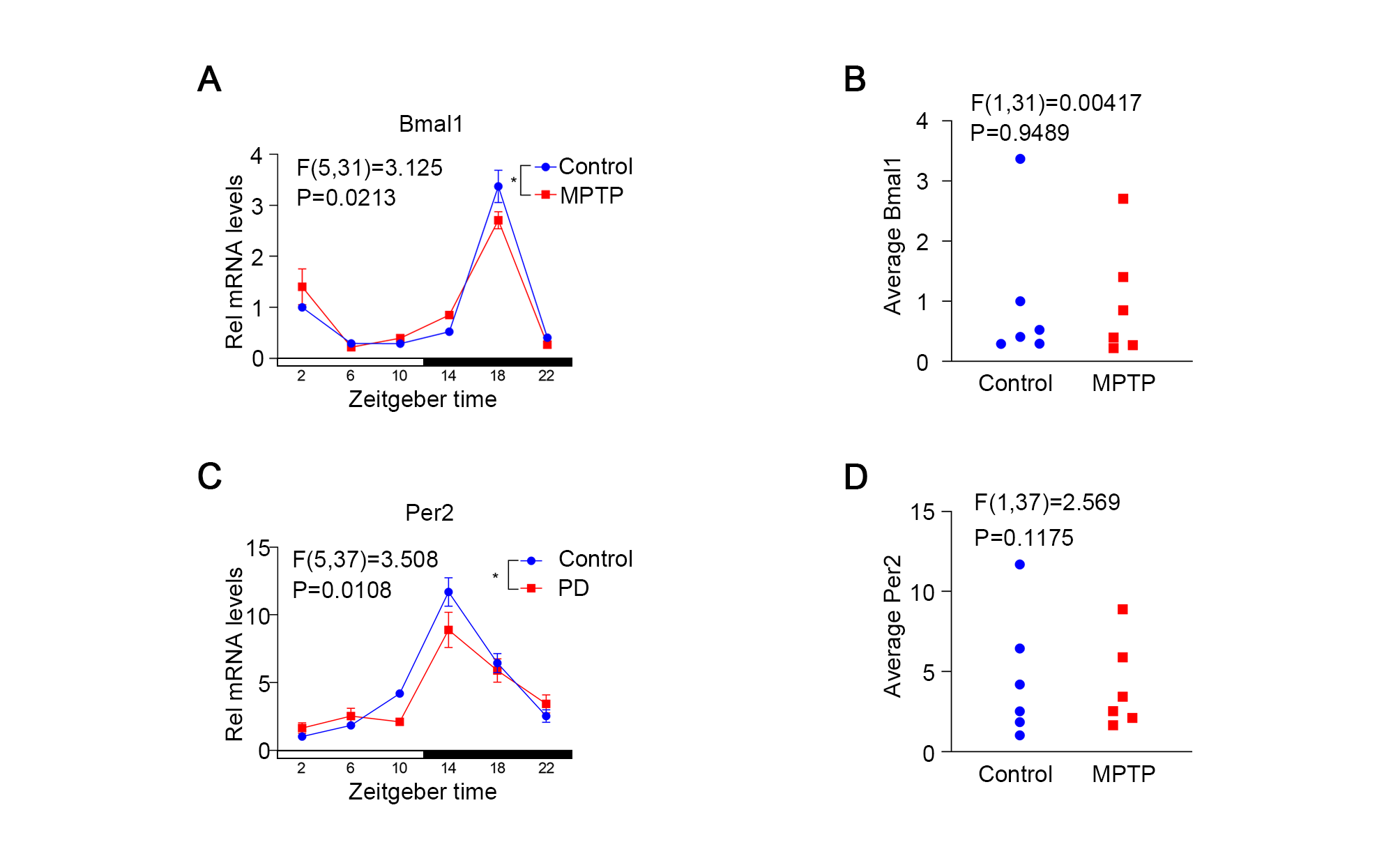

Supplement: Supplementary file 1 — Additional file 1: Figure S1. Aberrant diurnal Bmal1 and Per2 rhythm in the SN of MPTP-induced Mice. (A and C) The mRNA level of Bmal1 and Per2 in the SN was quantified using real-time PCR. (*p < 0.05, two-way ANOVA test, interaction between time and genotypes). (B and D) The average values of Bmal1 and Per2 over the course of the day were calculated. (two-way ANOVA test, interaction between genotypes). n = 3–5 for each time point. Data were presented as mean ± SEM. [file 12974_2022_2494_MOESM1_ESM.tif]
